# Infusum Juniperi Compositum

**Published:** 1853-10

**Authors:** 


### MATERIA MEDICA AND THERAPEUTICS.

*Infusum Juniperi Compositum*—Mr. Procter says: Having had occasion to prepare a variety of diuretics, for a member of my family affected with valvular disease, attended by dropsical effusion into the pleura and pericardium, none of them proved so successful as the infusion of Juniper berries with cream of tartar. The necessity of making the infusion every day, led to the adoption of the following formula, which was afterwards used with much satisfaction.

Take of Juniper berries, thoroughly bruised, four ounces.
Holland gin, - - four fluid ounces.
Boiling water, - - twelve fluid ounces.

Pour the boiling water over the bruised juniper, add the gin, mix them and let them macerate for twelve hours. The whole should then be thrown on a cotton cloth and expressed, and sufficient water added to the dregs and pressed out to make the infusion measure a pint.

The preparation thus made has an opaque brown color, and a decided odor and taste of the berries. When desirable, it may be made more active by rubbing one drachm of the oil of juniper with the bruised berries, then pouring the gin upon them, and after macerating an hour, adding the boiling water, and continuing the maceration for eight or ten hours longer. The liquid is then separated by expression as above. When made by the last method, the infusion contains more of the oleoresin of juniper in suspension, and is much less agreeable to the taste, but is more active and efficient. When the patient is troubled with gastric irritability, the first infusion is more appropiate. Whichever may be employed, the dose is from a tablespoonful to a wine glassful, mixed with a teaspoonful or more of cream of tartar, three times a day regularly. It is much better in prescribing juniper berries and cream of tartar for a patient, to direct the salt to be kept separate from the berries, and administered by measured doses with the infusion after it is made; because, when they are infused together, a large portion of the bitartrate crystallizes among the juniper dregs as the infusion cools, and is lost by straining the infusion.—*Journal of Pharmacy.*

